# Tumor Glucose and Fatty Acid Metabolism in the Context of Anthracycline and Taxane-Based (Neo)Adjuvant Chemotherapy in Breast Carcinomas

**DOI:** 10.3389/fonc.2022.850401

**Published:** 2022-03-31

**Authors:** Anna Mária Tőkés, Stefan Vári-Kakas, Janina Kulka, Beáta Törőcsik

**Affiliations:** ^1^ 2nd Department of Pathology, Semmelweis University Budapest, Budapest, Hungary; ^2^ Department of Computers and Information Technology, Faculty of Electrical Engineering and Information Technology, University of Oradea, Oradea, Romania; ^3^ Department of Biochemistry, Semmelweis University Budapest, Budapest, Hungary

**Keywords:** breast carcinoma, neoadjuvant and adjuvant chemotherapy, anthracycline, taxane, glucose and lipid metabolism

## Abstract

Breast cancer is characterized by considerable metabolic diversity. A relatively high percentage of patients diagnosed with breast carcinoma do not respond to standard-of-care treatment, and alteration in metabolic pathways nowadays is considered one of the major mechanisms responsible for therapeutic resistance. Consequently, there is an emerging need to understand how metabolism shapes therapy response, therapy resistance and not ultimately to analyze the metabolic changes occurring after different treatment regimens. The most commonly applied neoadjuvant chemotherapy regimens in breast cancer contain an anthracycline (doxorubicin or epirubicin) in combination or sequentially administered with taxanes (paclitaxel or docetaxel). Despite several efforts, drug resistance is still frequent in many types of breast cancer, decreasing patients’ survival. Understanding how tumor cells rapidly rewire their signaling pathways to persist after neoadjuvant cancer treatment have to be analyzed in detail and in a more complex system to enable scientists to design novel treatment strategies that target different aspects of tumor cells and tumor resistance. Tumor heterogeneity, the rapidly changing environmental context, differences in nutrient use among different cell types, the cooperative or competitive relationships between cells pose additional challenges in profound analyzes of metabolic changes in different breast carcinoma subtypes and treatment protocols. Delineating the contribution of metabolic pathways to tumor differentiation, progression, and resistance to different drugs is also the focus of research. The present review discusses the changes in glucose and fatty acid pathways associated with the most frequently applied chemotherapeutic drugs in breast cancer, as well the underlying molecular mechanisms and corresponding novel therapeutic strategies.

## Introduction

Neoadjuvant systemic therapy (NeST) is routinely used to treat breast cancer, with significant geographical variation in its use across different countries (1, 2). When introduced, NeST was used mostly for the treatment of patients with locally advanced or inoperable breast cancer aiming to reduce the tumor size, enabling post-therapy breast-conservation surgery. Currently, the role of NeST has expanded and includes different patient groups with early-stage, operable breast cancer with the following benefits: checking the sensitivity of a tumor to therapy *in vivo*, reduction of postoperative complications such as lymphoedema, prolonged disease-free survival, and improved cosmetic outcomes (1, 2).

Since the first clinical trial (The National Surgical Adjuvant Breast and Bowel Project B-18 trial) from 1997 that compared the prognostic value of neoadjuvant and adjuvant use of the same chemotherapy regimen, results of several other clinical trials have been published reporting no significant differences in the long-term outcomes for early-stage and locally advanced breast cancer with either approach (1, 3, 4). Meanwhile, several studies confirmed and validated pathological complete response (pCR) to NeST as a strong predictive factor of favourable long-term outcomes. In contrast, patients with a significant residual cancer burden have a higher risk of distant metastases (5, 6). In unselected breast cancer patient groups treated with NeST approximately 20-26% of the cases can achieve pCR but this rate is significantly higher in HER2-positive and triple-negative breast cancer (TNBC) subtypes achieving pCR rates of up to 50-60% (7–9). Based on these data it is clear that a fraction of TNBC and HER2-positive breast carcinomas does not achieve pCR and some hormone receptor (HR)-positive/HER2-negative patients respond to NeST. It is also clear that new factors like tumor heterogeneity, tumor metabolic diversity have to be considered for future oncological strategies in breast cancer treatment (10).

Personalized therapeutical approaches in both the neoadjuvant and adjuvant setting play more and more important role in breast cancer management. Adding immunotherapies [anti-PD-L1 (durvalumab) or anti-PD-1 (pembrolizumab)] or PARP-inhibitor (talazoparib) to standard chemotherapy may improve the rate of pCR in TNBC and women with germline BRCA mutations (11, 12).

The classification of most frequently used chemotherapeutic drugs in neoadjuvant and adjuvant settings - mostly DNA alkylating, antimicrotubule, immunologic and hormonal agents, and antimetabolites - are presented in **Table 1**. The applied neoadjuvant chemotherapy regimens in breast carcinoma cases may differ across countries. Most commonly, anthracycline (AC) (doxorubicin or epirubicin) is administrated in combination or sequentially with taxanes (paclitaxel or docetaxel). To enhance cytotoxicity AC is frequently used in combination with cyclophosphamide with or without fluoropyrimidines (13).

**Table 1:** Classification of most frequently used drugs in patients diagnosed with breast carcinomas based on the mode of action.

Category of chemotherapeutic drugs	Mechanism of action	Chemotherapeutic agents		
Antimetabolite	Inhibit enzymes involved in DNA synthesis and DNA replication, cell cycle regulation	Antifolate (dihydrofolate reductase inhibitor)	Methotrexate	
		Nucleoside analog	Gemcitabine, Capecitabine, 5-flurouracil	
		Topoisomerase II inhibitor	Doxorubicin, Metoxantrone	
		Kinase inhibitor	Palbociclib, Ribociclib,	
Antimitotic	Inhibit mitosis by binding to micotubules	Synthetic	Ixabepilone	
		Alter microtubule function/Natural	Paclitaxel, Docetaxel, Vinblastine	
DNA alkylation	Intercalate with DNA, form crosslink	Platinum-based	Cisplatin, Carboplatin	
		Nitrogen mustard	Cyclophosphamide	
Hormonal	Blocking the hormone-receptor binding on cancer cells	GnRH	Goserelin	
		Antiestrogen	Receptor inhibitor	Fulvestrant, Tamoxifen
			Aromatase inhibitor	Examestane, Letrozole, Anastrozole
		Progesterone	Megestrol acetate	
ERBB2+ targeting agents	By attaching itself to the HER2 receptors block cells from receiving growth signals	Monoclonal antibodies	Trastuzumab, Pertuzumab	
		Antibody-Drug Conjugate	Ado-Trastuzumab	
Immunotherapies	Immune Checkpoint Inhibitors	Atezolizumab, Pembrolizumab		

Considering the breast carcinoma subtypes in the UK the most commonly prescribed neoadjuvant chemotherapy regimes in breast cancers are: AC-containing combinations of fluorouracil, epirubicin and cyclophosphamide (FEC) with docetaxel/trastuzumab/pertuzumab for HER2-positive disease and FEC-docetaxel for HER2-negative disease (2).

**Figure 1:**
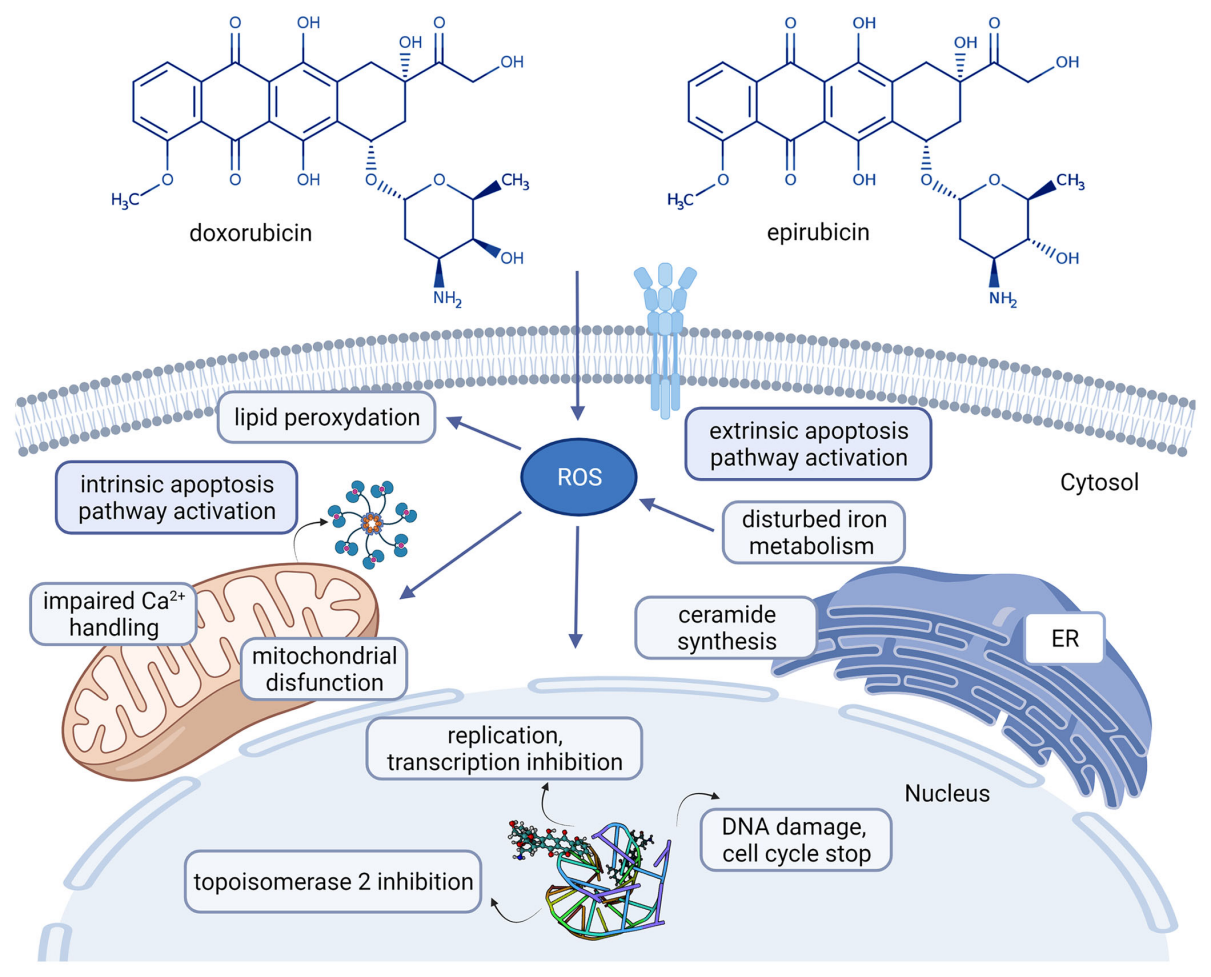
Chemical structures of the clinically used anthracyclines in breast cancer treatment and their mechanism of action [doxorubicin: CHEBI:28748, epirubicin CHEBI:47898 (14), adriamycin-DNA interaction: PDB ID 6KN4 (15)]. Created with BioRender.com. Agreement number: UB23MRCQE9.

*Anthracylines* are broadly used as anticancer agents. The first AC described was daunorubicin from which several derivatives were developed for clinical use, including doxorubicin (DOX) and epirubicin (**Figure 1**) (14).

Despite their efficacy treatment resistance (as a multifactorial clinical issue) and toxicity (especially cardiotoxicity) have to be considered in each patient before prescription (16, 17).

Several trials were and are designed to compare the effectiveness between ACs and/or taxane as neoadjuvant chemotherapy (1, 18) and repeatedly concluded that pCR is a prognostic marker of significantly higher disease-free survival and overall survival (4, 18). The rate of pCR highly depends on patients and drug selection but generally is lowest for hormone receptor (HR)-positive, HER2-negative tumors and increases approximately additively for HER2-positive/HR-negative tumors with considerable differences between different studies.

The Collaborative Trials in Neoadjuvant Breast Cancer group analyzing the pCR association with long-term outcome have found that the highest pCR was achieved in HER2-positive/HR-negative breast cancer (50.3%) after administration of trastuzumab followed by TNBC (33.6%) and by grade 3 HR-positive/HER2-negative breast cancer with a pCR rate of (16.2%) (1, 19).

To understand how the metabolic signature of a tumor is associated with drug resistance, first we have to understand the mode of action of that drug. ACs intercalate with DNA bases and stop the activity of polymerases blocking in this way the DNA and RNA synthesis (20). Recent studies demonstrated that after diffusion of AC through the plasma membrane, cytosolic AC forms a complex with proteasome that is later transferred into the nucleus, a process requiring ATP (21). ACs were also found to induce cytotoxicity through topoisomerase II (TOPOII) inhibition (block the catalytic activity of TOPOII, contributing to inhibition of DNA replication) (20). Free radicals damaging cell membranes as well as DNA and proteins are described under DOX treatment. The potential involvement of free radical generation in the cytotoxicity of AC is complex and not completely understood (16). ACs can also induce cell death mediated by ceramide. In 2012 Denard B et al. described that treatment of cancer cells with DOX-induced ceramide synthesis (a bioactive sphingolipid, N-acylsphingosine resulting from four consecutive reactions from the precursors palmitoyl-CoA and serine) (22). The schematic mechanism of actions of ACs is presented in **Figure 1**.

As detailed above ACs act on different pathways to induce tumor cell death. Accordingly, tumor resistance to ACs is also a multifactorial problem that involves several and diverse mechanisms. The main resistance mechanisms are (16): 1. PGp-dependent and PGp-independent multidrug resistance, often associated with upregulation or amplification of the *MDR1* gene that encodes PGp. 2. DNA repair mechanisms. Spencer DM et al. demonstrated that nucleotide excision repair and homologous recombination are the most important mechanisms involved in DNA repair after ACs treatments (23). 3. Altered TOPOII activity. Mutation, abnormal expression of TOPOIIα subunit, suppression of TOPOII-mediated apoptotic signaling are associated with clinical resistance to ACs (24). 4. Cancer stemness. Several studies described a direct link between resistance to ACs and cancer stemness (25). 5. Metabolic changes associated with resistance.

**Figure 2:**
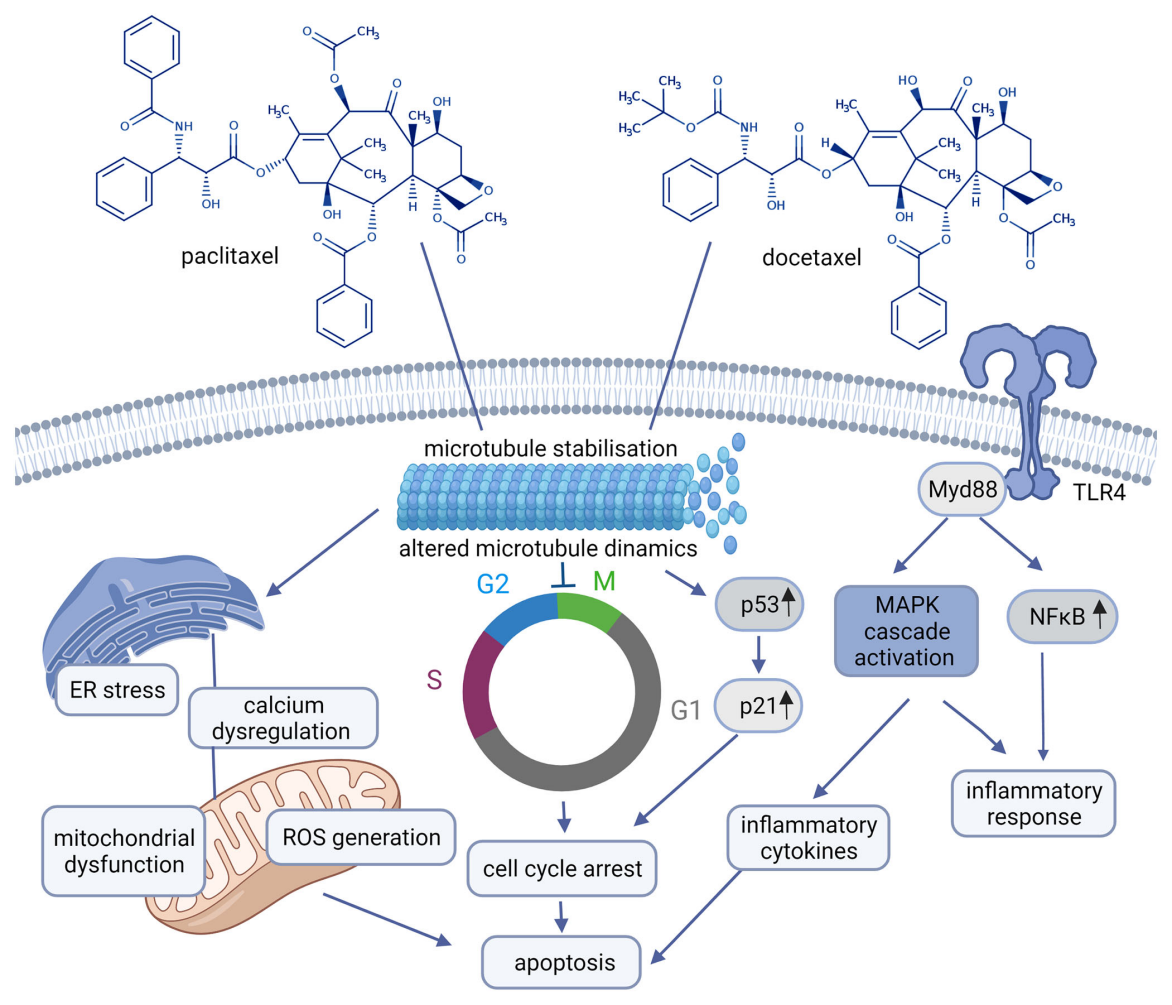
Chemical structures of paclitaxel, docetaxel and their mechanism of action (paclitaxel: CHEBI:45863, docetaxel: CHEBI:4672) Created with BioRender.com. Agreement number: UY23MRDFCE.

*Taxanes* as microtubule-stabilizing cancer drugs were introduced in the treatment of breast carcinomas in the 1990s. Taxanes (paclitaxel (PTX) and docetaxel (DTX)) suppress microtubules involved in a variety of cellular processes like cell division, signaling, migration by binding to the β-subunit of the tubulin heterodimer (26). PTX was the first discovered member of the taxane family. DTX differs structurally (see **Figure 2**) and functionally from PTX (14, 27).

Due to drug resistance observed after PTX and DTX treatment, cabazitaxel (CBZ) as a novel taxane drug

was developed and approved by the FDA in 2010 and used as a second-line treatment for metastatic castration-resistant prostate cancer patients (28).

Though the initial response to taxanes is impressive, the resistance to these drugs is frequently observed and is supposed to be coordinated by: 1. alterations in tubulin, 2. proteins that interact with microtubules, 3. efflux pumps, 4. apoptotic proteins, 5. signal transduction pathways, 6. tumor metabolism. Accordingly, strategies to overcome resistance to PTX and DTX include inhibition of the efflux pumps, the use of novel taxane not interacting with drug efflux pumps, regulation of apoptotic and signal transduction pathways, and inhibition of metabolic pathways involved in drug resistance (28, 29). Tubulin is associated with taxane resistance in different ways: 1. mutation in tubulin (high number of point mutations in tubulin have been found in different cell lines selected for paclitaxel resistance analyzes), 2. changes in the expression of tubulin isomers, 3. several proteins that bind to tubulin like: microtubule associated protein 4. posttranslational modifications of tubulin like phosphorylation, acetylation, tyrosination etc. (30–32).

How the sequence order of ACs and taxanes used in neoadjuvant settings influence the pCR rate is under considerable debate. A recent study showed that sequence order did not influence the primary endpoint of pCR rate (19% for AC-Taxane vs. 21% for Taxane-AC) and pCR rate was higher in patients with TNBC (32% vs. 13% in hormone-positive cancers) (33).

Our knowledge about the impact of different AC-derived therapeutic drugs on the metabolic pathways in different cancers is mostly unknown. Achkar IW et al. in a very impressive study have analyzed the metabolic pathways affected by DOX treatment in chicken embryo tumor models (in ovo). By using two mass-spectrometry based platforms (broad metabolic profiling HD4 platform, and the Lipdyzer complex lipid platform) they finally have found that after DOX treatment statistically significant alteration in 127 metabolites (affecting the metabolism of amino acids, carbohydrates, cofactors and vitamins as well as nucleotides and lipids) can be documented on HD4 platform. Whereas on lipid platform they observed statistically significant decrease in 96 lipid molecules. Their results clearly indicate that DOX treatment generates metabolic rewiring (34).

Related to paclitaxel a study performed on human breast carcinoma cell lines demonstrated significant differences in 31 metabolites such as fructose-6-phosphate, citric acid, glycerophosphoinositol and glycerol 3-phosphate when compared metabolic alterations in paclitaxel treated MCF-7 cell lines to untreated ones (35).

Dysregulated cancer metabolism has been increasingly associated with acquired resistance to chemotherapeutic treatment, but no clear data are presented about how metabolic pathways are activated following oncological treatment. In addition to glycolysis that remains favored in resistant cancers undergoing AC treatment, experimental evidences also involve pentose phosphate pathway, fatty acid metabolism, nucleotide synthesis etc. However, there is an urgent need to analyze the complex system of tumor metabolism as the most pieces of evidence are often based on a single pathway without alternative explanation (34, 36).

Beside dysregulated metabolism observed in several cancer types, metabolic reprogramming and metabolic heterogeneity play critical role in carcinogenesis and in acquired resistance to different chemotherapeutic agents. As cancer cells with different metabolic profiles may not respond similarly to anticancer treatment current studies suggest that metabolic heterogeneity needs to be integrated in routine practice to precisely predict breast cancer response to different treatments (37). It is a further challenge in cancer therapy to consider the complex system of inter- and intra-tumour metabolic heterogeneity. Considering just the increased glycolysis as a hallmark of dysregulated metabolism it is well documented that differences in glucose utilization are not only seen between cancer cells, but also within other cells comprising the tumors (38).

Of the relatively few studies demonstrating that chemotherapy agents used in the current treatment regimens in breast carcinomas cause metabolic reprogramming in cancer cells Desbats MA et al. presented that ACs induce increased glycolysis, GLUT1 and glutaminase level whereas taxanes induce elevation in glycolysis, in lactate dehydrogenase A (LDHA), pyruvate kinase M (PKM2), pyruvate dehydrogenase kinase 2 (PDK2) (inhibitor of pyruvate dehydrogenase complex), glutamine uptake, fatty acid synthase (FASN), mitophagy, mitochondrial mass and oxidative phosphorylation (OXPHOS) (39).

## Glucose Metabolism

*Glycolysis* is the major pathway of glucose metabolism occurring in the cytosol of the cells by which glucose is metabolized to pyruvate. Considering the aerobic and anaerobic conditions occurring in tumors and different organisms pyruvate transportation may follow two different pathways (36, 40).

Under aerobic conditions, pyruvate is transported into mitochondria, where it is converted to acetyl-CoA. Acetyl-CoA reacts with oxaloacetate to form citrate that later enters into the tricarboxylic acid cycle (TCA) and generates NADH and FADH_2_ (reducing equivalents) (41). These reducing equivalents later enter the electron transport chain leading to the production of 36-38 ATP per molecule of glucose.

Under anaerobic conditions, pyruvate instead of entering mitochondria follows a different pathway. In cytosol, the cytosolic enzyme lactate dehydrogenase (LDH) converts pyruvate to lactate. This reaction also allows for the regeneration of NAD^+^ (the primary oxidizing agent of glycolysis) from NADH (42).

Of the other pathways of carbohydrate metabolism (gluconeogenesis, glyoxylate cycle - typical for plants -, biosynthesis of oligosaccharides and glycoproteins, pentose phosphate pathway-PPP) the PPP pathway was described as highly affected in different tumor types (43).

**Figure 3:**
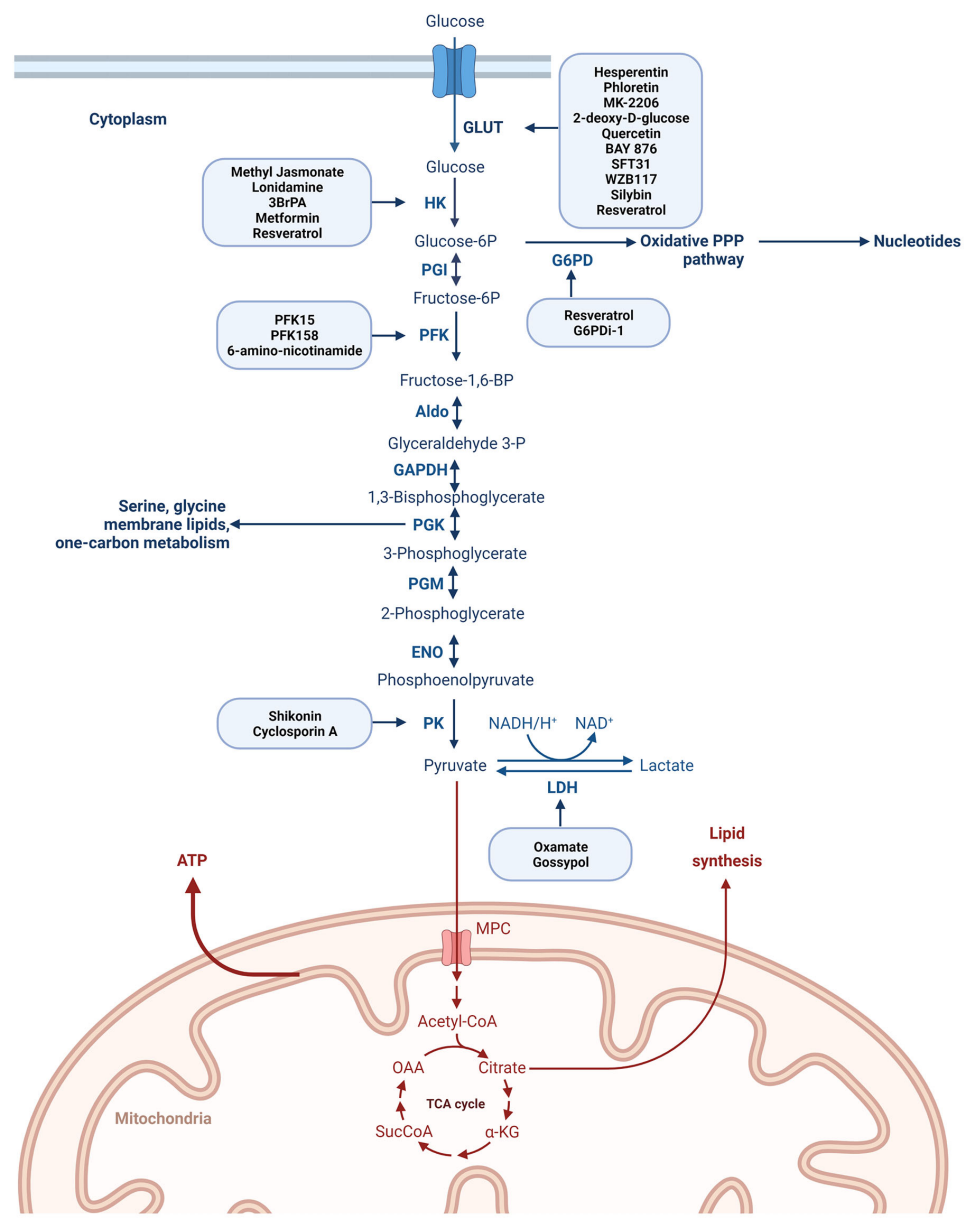
Degradation of glucose *via* the glycolytic pathway with the most representative enzymes playing a role in the process of glycolysis and with the main drugs described to inhibit different steps of the glucose metabolism. Created with BioRender.com. Agreement number: QP23MRDLKQ.

Glycolysis is controlled by the following ten enzymes and enzymatic reactions (44) (**Figure 3**): 1. Hexokinase-HK (transfers a phosphoryl group from ATP to glucose to form glucose-6-phosphate-G6P), 2. phosphoglucose isomerase-PGI (converts G6P to fructose-6-phosphate-F6P), 3. phosphofructokinase-1-PFK-1 (phosphorylates F6P to fructose-1,6-bisphosphate-FBP), 4. aldolase (cleaves FBP to form the two trioses, glyceraldehyde-3-phosphate-GAP and dihydroxyacetone phosphate-DHAP), 5. triose phosphate isomerase-TPI (converts DHAP to GAP), 6. glyceraldehyde-3-phosphate dehydrogenase-GAPDH (catalyzes the reversible oxidative phosphorylation of GAP, and forms the first high-energy intermediate, 1,3 bisphosphoglycerate -1,3 BPG) 7. phosphoglycerate kinase-PGK (generates 3-phosphoglycerate-3PG and the first ATP) 8. phosphoglycerate mutase-PGM (converts 3PG to 2-phosphoglycerate -2PG), 9. enolase (dehydrates 2PG to phosphoenolpyruvate-PEP, 10. pyruvate kinase-PK (couple the free energy of PEP hydrolysis to the synthesis of ATP to form pyruvate).

Glycolysis depends on a constant supply of glucose that enters by two different types of membrane- associated carrier proteins. Na^+^-glucose cotransporters (SGLTs) are present in the small intestine and the kidney, while passive transport occurs *via* ubiquitous glucose transporters, the GLUT family (45, 46).

The GLUT family consisting of 14 members facilitates the uptake of glucose through the cell membrane and plays crucial role in glycolysis (36, 46, 47). An increase in GLUT1–6 and 12 was reported in breast cancer (46, 48) and glucose uptake by GLUT1 is considered an important mechanism in breast carcinogenesis and plays an important role in the early phase of breast cancer development (49). Upregulation of GLUT1, 3, and 4 is associated with cancer resistance and inhibition of GLUT may sensitize the anticancer effect of chemotherapeutic drugs (50, 51). Several inhibitors of GLUT1 are reported in the literature and a non-exhaustive list is presented in **Figure 3**. Phloretin, a GLUT1 inhibitor re-sensitized colon and breast cancer cells to daunorubicin’s anticancer activity and apoptosis-inducing effects (51). GLUT1 inhibitors -WZB117 and SFT-31- inhibit cell proliferation and promote apoptosis in breast cancer cell lines and moreover it was shown that WZB117 increases the effectiveness of radiation (52). Another study presented that the combination of MK-2206 and WZB117 exerts a synergistic cytotoxic effect against breast cancer cells (53). BAY-876, a selective GLUT1 inhibitor analyzed on TNBC cell lines decreased glucose uptake (54). 2-deoxy-D-glucose (2-DG) (phosphorylated by hexokinase but not metabolized further) competes with glucose for binding GLUT reducing in this way glucose uptake in the MDA-MB-231 TNBC cell line (55).

As it is presented in **Figure 3** each reaction in glycolysis is catalyzed by its own enzyme. Many of these key glycolytic enzymes are highly expressed in different cancers and correlated with chemoresistance.

A non-exhaustive list of drugs targeting glucose metabolism are presented in **Table 2**.

**Table 2:** Non-exhaustive list of drugs targeting glucose metabolism.

Target	Drug name	Drug effect	References/trial number
GLUT	Hesperentin	Impairs glucose uptake and inhibits proliferation of breast cancer cells.	(56)
	Phloretin	Not fully elucidated	(55)
	MK-2206	pan-Akt inhibitor	(57)
	2-deoxy-D-glucose (2-DG)	2-DG competes with glucose for uptake into cells via the GLUT	(58)
	Quercetin	Suppresses the mobility of breast cancer by suppressing glycolysis through Akt-mTOR pathway	(59)
	BAY 876	Impairs the growth of a subset of TNBC cells displaying high glycolytic and lower oxidative phosphorylation (OXPHOS) rates	(54)
	SFT31	Inhibit cell proliferation and promote apoptosis in breast cancer cell lines	(41)
	WZB117	Inhibit cell proliferation and promote apoptosis in breast cancer cell lines	(41)
	Silybin	Counteracts doxorubicin resistance by inhibiting GLUT1 expression.	(75)
	Resveratrol	Suppresses cancer cell glucose uptake by targeting ROS-mediated hypoxia-inducible factor-1α activation. Enhances chemosensitivity of doxorubicin	(60)
HK	Methyl Jasmonate	Detaches hexokinase from the voltage-dependent anion channel	(61)
	Lonidamine	Affects DNA repair as well as cellular acidification	(62)
	3BrPA	Enhances drug accumulation by inactivating ABC transporters. Induce autophagy.	(63, 64)
	Metformin	Not fully elucidated its action in breast carcinomas Regulates AMP kinase, target stem cells	(65)
	Resveratrol	Decreases the cell viability and glucose consumption	(66)
PFK	PFK15	Apoptosis; cell cycle arrest in G0/G1 phase	(67)
	PFK158	Apoptosis; and increased ROS	(67)
	6-amino-nicotinamide	Decrease aldehyde dehydrogenase (ALDH) activity.	(41)
G6PD	Resveratrol	Decreases the cell viability and glucose consumption	(9, 41)
	G6PDi-1	More effectively inhibits G6PD	(68)
PK	Shikonin	Can inhibit the activities of DNA topoisomerases, inhibits the activity of pyruvate kinase M2 (PKM2)	(69)
	Cyclosporin A	Regulate the expression and activity of PKM2	(70)
LDH	Oxamate	Induce apoptosis in vitro, and reduces tumor growth in vivo	(71)
	Gossypol	Contradictory results	(72)

### Hexokinase II (HKII)

HKII is considered as the first rate-limiting enzyme in the glycolytic pathway (73). HKII was shown to be upregulated in breast cancer tissues and found to contribute to PTX resistance (74, 75). Even if the major role of GLUT-1 and HKII in cellular metabolism is highly documented their expression after DOX or taxane treatment has not been systematically evaluated. Based on the studies of Zhou R et al. several chemotherapeutic agents may have a significant and direct effect on GLUT-1 and HKII expression (76). Of the HKII inhibitors 3-bromopyruvate (3-BrPA), and lonidamine (LND) have been used in preclinical experiments (36). 3-BrPA enhances drug accumulation by inactivating ABC transporters restoring the cytotoxic effects of DOX. It was shown that 3-BrPA used in combination with DOX significantly inhibited the growth of subcutaneous tumors in multiple myeloma mice (36, 63). The anticancer effect of 3-BrPA was also demonstrated on hepatocellular carcinoma both in *in vitro* and in *in vivo* studies and consequently, this drug has been approved by the FDA (63). Inhibition of HKII by LND enhanced the effects of DOX in rituximab-resistant lymphoma cell lines (RRCL) and used in combination with platinum and paclitaxel presented a good tolerability (77, 78).

However metformin, one of the most frequently used drugs in the treatment of type II diabetes mellitus demonstrated antitumor activity combined with or following other therapeutic agents *in vitro* and *in vivo*. The detailed mechanism of action of metformin in tumors is not fully elucidated but based on recent studies is mediated through regulation of AMP kinase (AMPK)/mammalian target of rapamycin (mTOR) and insulin/IGF-1 signaling pathways (65). Studies present several aspects of metformin activity in breast carcinomas like metformin downregulation of protein and lipid synthesis, modulation of mitochondrial respiration, induction of stem cell death etc. have been presented and discussed. The authors also outline that many of these anti-cancer effects are molecular subtype-specific being most potent in triple-negative breast carcinomas (79, 80). Nonetheless a study performed on 320,000 persons diagnosed with incident diabetes mellitus has not found any association between metformin treatment and the incidence of major cancers (81).

### *Glucose*-6-Phosphate Dehydrogenase (G6PD)

The first step of the pentose phosphate pathway (PPP) is catalyzed by the G6PD enzyme. A relatively high number of studies have interrogated the role of G6PD in different cancer types but how the overexpression of G6PD contributes to the development of different tumors is largely unknown (82). Yang HC et al. in a very recent study have demonstrated that deregulated G6PD status and oxidative stress are highly related and are involved in cancer progression (83). Another study analyzing MDA-MB-231 cells implanted as orthotopic xenografts has found that the loss of G6PD modestly decreased primary site growth and the ability of breast cancer cells to colonize the lung (84). A relatively high number of G6PD inhibitors were documented in the last years but sometimes with contradictory results. 6-aminonicotinamide, was found to decrease mammosphere formation and aldehyde dehydrogenase activity (85, 86). Ghergurovich JM et al. presented that the most known G6PD antagonist, dehydroepiandrosterone, does not inhibit robustly G6PD in cells and as a consequence they have identified a small molecule (G6PDi-1) that more effectively inhibits G6PD and later the PPP pathway (68).

### Phosphofructokinase (PFK)

Our understanding of PFK enzymes and their roles in cancer has developed significantly in the last years. The formation of fructose-1,6-bisphosphate from fructose-6-phosphate is catalyzed by PFK-1 in an irreversible step and therefore PKF-1 is considered as one of the most important rate-limiting enzyme in the process of glycolysis and is regulated by several effectors like fructose 2,6-bisphosphate (F2,6-BP), AMP and ATP. Moreover F2,6-BP levels are strongly associated with the bifunctional enzyme 6-phosphofructo-2-kinase/fructose-2,6-bisphosphatase (PFK-2, PFKFB) which display tissue-specific pattern. Four known isozymes of PFKFB are described (PFKFB1, PFKFB2, PFKFB3 and PFKFB4) with different kinase-to-phosphatase activities (87).

Of the PFKFB family members, PFKFB3 and PFKFB4 in particular, are overexpressed in breast cancers and in numerous other malignancies (87). PFKFB3 presents the highest kinase activity among the four isoforms and its inhibition resulted in the suppression of the growth of tumor cells by downregulating the glycolytic flux (74, 88).

Considering that standard chemotherapy inevitably is associated with the development of chemoresistance, the observation made by Kotowski K. et al. that PFKFB3 inhibition therapy concurs with carboplatin and paclitaxel in therapy-resistant cell lines of gynecological cancers to reduce tumor weight presents an important step in further therapeutic approach (87). Resveratrol (a natural product found in various plants) nowadays is also considered as a potential anti-tumoral drug that reduces glucose metabolism and viability in cancer cells. Gomez LS et al. have demonstrated that resveratrol decreases cell viability, glucose consumption and ATP content in the human breast cancer cell line MCF-7 (66).

### Phosphoglycerate-Kinase (PGK)

Phosphoglycerate-kinase (PGK) catalyzes the first substrate-level phosphorylation in glycolysis while producing 3-PG. PGK1 activity is highly responsible for maintaining energy homeostasis and serine biosynthesis. Clinically, PGK1 was overexpressed in many types of tumors. The exact molecular mechanisms for PGK1-involved drug resistance are not fully clarified but were found to be upregulated in radioresistant astrocytomas and cisplatin-resistant ovarian cancers. Moreover, inhibition of PGK1 increased the sensitivity of gastric adenocarcinoma cells to chemotherapeutic drugs 5-fluorouracil and mitomycin-C (89–91). Sun S et al. by analyzing PGK1 at protein and mRNA level in breast carcinoma cases have found that high PGK1 expression is associated with worse overall survival and concluded that PGK1 may be considered as a predictive biomarker of chemoresistance to paclitaxel treatment in breast cancer (92).

### Enolase (ENO)

Proteomics profiling revealed high enolase-1 (ENO-1) expression in ER+ breast carcinomas. The high ENO-1 status was correlated with poor prognosis and with positive nodal status (93). Qian X et al. by analyzing gastric cancers have found that elevated levels of ENO-1 proteins (or downregulation of ENO-1 targeting miR-22) were associated with shorter overall survival and ENO-1 is a novel biomarker to predict drug resistance and overall prognosis in gastric cancer. Based on their data targeting ENO-1 by chemical inhibitors or upregulating miR-22 could be valuable to overcome drug resistance (94). The ENO functions in different cancers and as a potential cancer biomarker are summarized in a very recent study by Almaguel FA et al. (95).

### Pyruvate-Kinase (PK)

The conversion of phosphoenolpyruvate to pyruvate with generation of ATP is catalyzed by PK. Till now four PK isoforms are described: PKM1, PKM2, PKR, and PKL (96). Apart from regulating glucose metabolism, PKs are involved in the modulation of gene expression, cell cycle regulation and cell–cell communication (97). Both PKM1 and PKM2 protein expression showed high levels in TNBC and targeting PK inhibited the proliferation of TNBC MDA-MB-231 and MDA-MB-436 cells by involving the NFκB signaling pathway (98). In a study involving 296 invasive breast carcinoma samples a correlation between PKM2 expression and the prediction of chemosensitivity to epirubicin and 5-fluorouracil was demonstrated (99). Moreover, in ER+ breast carcinoma models using MCF-7 and T47D cells PKM2 enhanced chemotherapy resistance by promoting aerobic glycolysis (100). It was also shown that PKM2 expression correlated mostly with cisplatin resistance in breast carcinomas (74).

### Lactate Dehydrogenase (LDH) and Drug Resistance

A large amount of literature has been published regarding LDH expression in different cancers, its effect on cancer development and progression, and its diagnostic and prognostic significance. Lactate production is an important phenomenon in the cancer microenvironment and is used as a mechanism of tumor escape from the immune response.

LDH is a tetrameric enzyme composed of two different subunits LDHA (M) and LDHB (H) (encoded in humans by *LDHA* and *LDHB* genes, which can assemble into five different isoenzymes as LDH1 or LDHB (H4), LDH2 (M1H3), LDH3 (M2H2), LDH4 (M3H1), and LDH5 or LDHA (M4) and is involved in the conversion of pyruvate to lactate. Deregulated levels of LDHs have been reported in multiple tumors (101, 102). In cancer cells, LDHA plays an important role in rapid conversion of pyruvate to lactate, minimizing in this way the pyruvate entry into TCA cycle in the mitochondria (103). LDHA is also involved in tumor cells proliferation, angiogenesis, tumor cells invasion and migration (103–105).

Based on the review by Mishra D et al. inhibition of LDHA and LDHB is of great interest and is unlikely to cause any possible side effect (103).

Data presenting the role of LDHA in drug resistance described that in chondrosarcoma inhibiting LDHA increased cancer cell sensitivity to DOX (106), in breast cancer cells, led to re-sensitization to paclitaxel (107). A link between LDHA and paclitaxel resistance was described by Varghese E et al. (74). Oxamate, an inhibitor of LDHA, combined with paclitaxel-induced apoptosis in paclitaxel-resistant breast carcinoma (MDA-MB-435 and MDA-MB-231) cells by inhibiting cellular glycolysis (107).

Inhibition of LDHA by small molecule inhibitors or its knockdown by siRNAs or shRNAs decreases tumorigenicity (107). LDHA seems to be a safe therapeutic target, as patients with hereditary LDHA gene deficiency only show symptoms (exertional myoglobinuria) after strenuous exercise but not under ordinary circumstances (108). In accordance with gene deficiency, LDHA inhibition was not proven to be harmful to normal cells (109). Studies showed that LDHB is up-regulated in triple-negative breast cancer (110). Recently, a highly selective LDHB inhibitor was identified (111).

### PPP Pathway

In this pathway NADPH is generated by the oxidation of G6P *via* an alternative pathway to glycolysis. In PPP ribose-5-phosphate-R5P is synthesized as an essential precursor in nucleotide biosynthesis. PPP is made up of two branches: the oxidative and the non-oxidative. In the PPP oxidative phase, G6P is converted into ribulose 5-phosphate and CO_2_, leading to the synthesis of NADPH.The non-oxidative arm of the PPP composed of a series of reversible reactions generates pentose phosphates for ribonucleotide synthesis (112).

PPP activation has been described in different types of cancer and was associated with metastasis, angiogenesis, and response to chemotherapy and radiotherapy (113). Giacomini I et al. have found that in particular, the oxidative branch of PPP seems to be involved in cisplatin resistance (114). They also suggest that the possibility to selectively deliver into the same cancer cell an anticancer drug and a PPP inhibitor or drug affecting the glucose seems a strategy to overcome the problem of drug resistance (114). Goldman A et al. demonstrated that after taxane treatment metabolic reprogramming of breast cancer cells was observed and characterized by increased glycolytic and oxidative respiration and glucose flux through the PPP pathway (115). The association of PPP with cancer resistance to ACs has been described in several studies (16, 116). Polimeni M et al. (116) by comparing a DOX-resistant human colon cancer cell line (HT29-DX) with a DOX-sensitive (HT29 cells) have found increased PPP and G6PD activity in DOX-resistant cell lines (116).

### Citric Acid Cycle

Alternatively known as the tricarboxylic acid cycle (TCA) or Krebs cycle is a hub of the metabolic system. Acetyl-CoA, a common product of carbohydrate, fatty acid and amino acid metabolism enters TCA cycle that oxidizes the acetyl group of acetyl-CoA to two molecules of CO_2_ with the generation of three NADH, one FADH_2_ and one ATP equivalent GTP.

**Figure 4:**
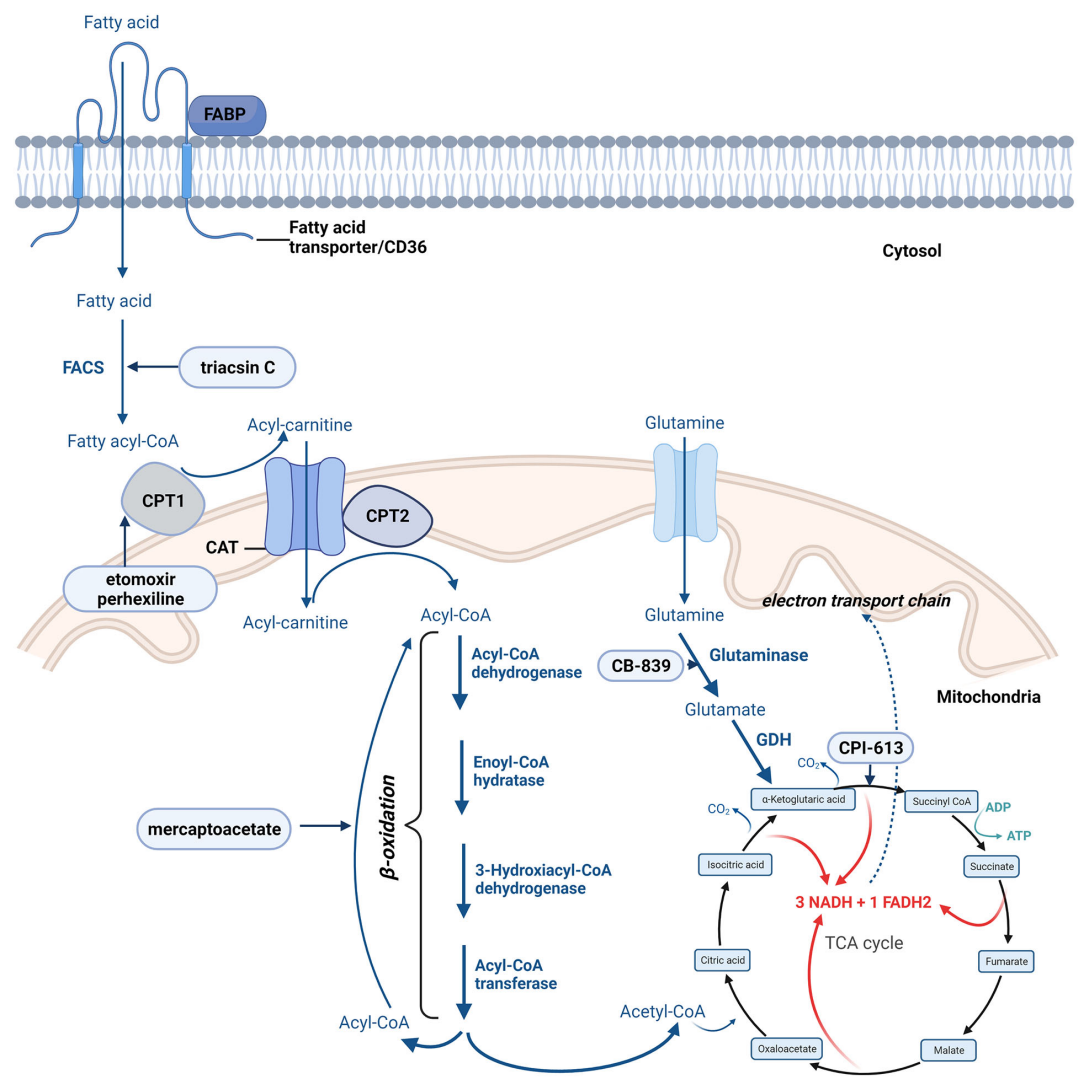
The most important steps of fatty acid oxidation. Created with BioRender.com. Agreement number: ZA23MRDTIU.

The main enzymes involved in TCA cycle are: 1. citrate synthase (catalyzes the condensation of acetyl-CoA and oxaloacetate to yield citrate), 2. aconitase (isomerizes citrate), 3. isocitrate dehydrogenase (catalyzes the oxidative decarboxylation of isocitrate to α-ketoglutarate with the coupled reduction of NAD^+^ to NADH and CO_2_ release), 4. α-ketoglutarate dehydrogenase (decarboxylates α-ketoglutarate to succinyl-CoA, releasing CO_2_ and NADH, 5. succinyl-CoA synthetase (converts succinyl-CoA to succinate with the coupled synthesis of a GTP), 6. succinate dehydrogenase (catalyzes the dehydrogenation of succinate yielding fumarate and FADH_2_, 7. fumarase (catalyzes the hydration of fumarates to yield malate) and 8. malate dehydrogenase (reforms oxaloacetate) (117) (**Figure 4**).

NADH and FADH_2_ are important products of citric acid cycle. Their reoxidation by O_2_ through the mediation of the electron-transport chain during OXPHOS completes the metabolic breakdown (117).

Several cancer types are characterized by drastic changes in TCA cycle enzymes compared to normal tissues. Accordingly components of the TCA cycle may be exploited therapeutically for the treatment of disease. Due to the importance of the TCA cycle in normal cell development, high toxicity of this approach have to be considered. By performing metabolomics profiling Ning Shen et al. have found significant changes in the TCA cycle and reactive oxygen species (ROS) related pathways in sensitive TNBC cells compared to resistant TNBC cells (118). Applying small molecule inhibitors to disturb the enhanced TCA for cancer treatment start to evolve CPI-613 (inhibiting both prolyl hydroxylases and α-ketoglutarate dehydrogenase complex- as a key regulator enzyme of TCA) is being tested in phase I and II clinical trials, as a single agent or in combination with standard chemotherapy to treat cancers (119). As many tumors utilize glutamine as a source for TCA cycle, suppression of glutaminolysis by small molecule inhibitors seems also an attractive approach to target tumors. CB-839 disrupts the conversion of glutamine to glutamate and alters TCA cycle, glutathione production, and amino acid synthesis (119).

### OXPHOS

Oxidative phosphorylation is an active metabolic pathway in many cancers and there is great interest in targeting mitochondrial OXPHOS in cancer. Evans KW et al. performed RNA sequencing from pre-treatment biopsies of TNBC patients who received neoadjuvant chemotherapy. They demonstrated that the top canonical pathway associated with worse outcome showed higher expression of OXPHOS signature. They also found that inhibiting OXPHOS may be a novel approach to enhance efficacy of several targeted therapies in TNBCs and have validated the antitumor efficacy of the combination of palbociclib, a CDK4/6 inhibitor, and IACS-10759 (a selective OXPHOS inhibitor that blocks complex I) *in vitro* and *in vivo* (120). Another study has established that TNBC cell lines (MDA-MB-468 and MDA-MB-231) were highly dependent on OXPHOS when compared to HR+ cell line MCF-7 (121). Considering the ubiquitous necessity of OXPHOS in healthy cellular metabolism, a major barrier to mitochondrial-targeted drugs is the need for cancer-cell selectivity.

## Future Challenges in Glucose Metabolism and Breast Cancer Treatment

The increased uptake of glucose, hyperactivated glycolysis and the accumulation of lactate are the main alterations in glucose metabolism associated with different tumor types. As future challenges the following questions must be addressed and discussed in more detail:

1. How the two frequently used ACs, DOX and epirubicin, generate distinct metabolic vulnerabilities in human breast cancer cells? This question is discussed in one recent study by McGuirk S et al. and they have found that in contrast to DOX-resistant cells, epirubicin-resistant cells present a drastic increase in OXPHOS and were more sensitive than DOX-resistant cells to treatment with phenformin. They have noticed that resistance to DOX was mostly associated with glutathione metabolism, whereas resistance to epirubicin to increased mitochondrial bioenergetic capacity (122). No similar data are available about the two frequently used taxanes (PTX and DTX) whether they elicit distinct primary metabolic vulnerabilities in human breast cancer cells.
2. Drug resistance is induced directly by one of the elements involved in the pathway (transporters, enzymes) or indirectly, through an overall increase of the glycolytic metabolism or both situations can occur? Marcucci F et al. in a recent review show that both situations can be present, and the upregulation of an individual enzyme is associated with enhanced overall elevation of the glycolytic metabolism (40).
3. What could be the importance of the fact that particular isoforms (ex. HK2, PFKFB isoform 3, PGK isoform 1 etc.) of the glycolytic enzymes expressed in tumor cells are involved in the induction of drug resistance and of the fact that some of these isoforms are associated with normal cells during embryonic development? (40).
4. How glucose-related metabolic changes are restored months or years after neoadjuvant therapy? Few studies address this question. According to a very recent study neoadjuvant chemotherapy worsens metabolic profile parameters (body mass index, total cholesterol, and fasting glucose) which are then recovered over 3 years. However, in the patients treated with neoadjuvant endocrine therapy there were no significant changes in fasting glucose and total cholesterol (123).
5. A hot topic in the oncological treatment is how selected metabolic inhibitors work alone or how they can be used in combination as potential treatments in breast cancer cases? How metabolic inhibitors used in combination with currently used drugs result in dose reduction of the chemotherapeutic agents known to be highly toxic? In a very recent study Draguet A et al. have evaluated the effect of selected metabolic inhibitors alone and in combination in two (MDA-MB-231 and MCF-7) breast cancer cell lines. Special attention was given to the analysis of metabolic inhibitors in combination with DOX. Their results are encouraging as the combination of CB-839 (glutaminase inhibitor) and Oxamate (lactate dehydrogenase inhibitor) and the combination of CB-839/Oxamate/D609 (a phosphatidylcholine-specific phospholipase C inhibitor) resulted in remarkable cell mortality and all the inhibitors improved the efficacy of DOX in cell lines. In addition, the same inhibitors improved the efficacy of DOX. Some of the metabolic inhibitors presented in their study like AZD3965 (a selective monocarboxylate transporter 1 inhibitor) are being evaluated in preclinical trials or in clinical trials (CB-839 - a glutaminase inhibitor) (124).

## Lipid Metabolism

Obesity is considered as a risk factor of breast cancer especially in post-menopausal women correlating with a diminished therapeutic response and with worse disease outcome (125). By analyzing obese vs. lean mice it was shown that decreased efficacy of DOX corresponds with alterations of lipid metabolism markers (126, 127).

Elevated and dysregulated lipid metabolism is a common hallmark of cells surviving neoadjuvant therapy in breast cancer patients (128). Studies also suggested that aberrant lipid metabolism plays an important role in cancer cells’ adaptation to treatment-induced cellular stress (129).

With the advent of new and more effective tools to study lipids is more and more recognized that lipids are central players in cancer biology (are essential builders for membranes, serve as fuel to the highly energetic process of uncontrolled tumor cell division and regulate numerous cellular functions) (130).

It is intriguing to analyse whether the breast cancer subtype defined by the transcriptome is reflected in the lipidome of breast cancer cells or whether is there any relationship between lipidomic profile and response to different therapies. Eiriksson FF et al. by performing liquid chromatography mass spectrometry (LC-MS) to analyze the lipidome of six breast cancer cell lines of different subtypes have found differences in lipidomes within the previously defined subtypes and concluded that subtypes defined by the transcriptome are also reflected in differences in the lipidome (131).

As it is presented in some of the recently published papers it would be also important to discuss in further reviews the utility of circulating lipid metabolites [to enhance the accuracy of known tumor markers or to distinguish tumors with early-stage vs. benign tumors (132)], the importance of lysophosphatidic acids (LPAs) as their role in different cancers is of great interest from therapeutic view and the role of the polyunsaturated fatty acids (PUFA). Recent studies analysing the role of the arachidonic acid (AA), a representative PUFA in different tumors have found a link between AA and macrophage function in ovarian cancer and have demonstrated that high level of AA is associated with poor clinical outcome in ovarian cancers (133). Another recent study has presented that in chemoresistant malignant pleural mesothelioma AA is a mediator of the adaptive response to pemetrexed (134)

Based on preclinical models lipid metabolism inhibition reversed the resistance of cancer cells to anticancer drugs suggesting that lipid metabolism play important role in drug resistance (129). The question is how, and at which step lipid metabolic changes contribute to drug resistance. Germain N et al. have suggested that at least the following changes in lipid metabolism may contribute to anticancer drug resistance: 1. lipid metabolism counteracts oxidative and ER stress-induced by anticancer drugs, 2. reduces metabolic stress and genotoxicity induced by anticancer drugs and contributes to the maintenance of drug-resistant cancer stem cells (129).

Pharmacological inhibitors have been developed for some of the enzymes of lipid metabolism and some of the compounds are used in combination with conventional therapies (129).

The dysregulation of lipid metabolism may occur at different steps of the metabolic process. The most frequent aberrations associated with drug resistance are overactivation of fatty acid oxidation, elevated fatty acid biosynthesis, aberrant accumulation of lipid droplets (LD) and changes in lipid composition of cell membranes (135).

Changes in lipid metabolism of resistant cells are considered treatment-specific and may include changes both in *de novo* lipogenic synthesis and/or lipolytic pathway (129). **Table 3** lists the most frequently used drugs in breast cancer treatment and the possible lipid metabolism pathway associated with resistance to different drugs.

**Table 3:** The most frequently used drugs in breast cancer treatment and the possible lipid metabolism pathway associated with resistance to different drugs.

Resistance to Drugs		Lipid Metabolism Reprogramming in Resistant Cells		
Drug	Drug Target	Pathway	Mechanism	Reference
Paclitaxel	Antimicrotubule agent	Increased lipolysis	High mRNA levels of CPT1B and FAO	(136)
Doxorubicin and Mitoxantrone	DNA binding and Topoisomerase II inhibitor	Increased lipogenesis	Increased FASN expression	(137)
Cisplatin	DNA binding	Increased lipogenesis	Increased FASN expression	(138)
Lapatinib	Inhibitor of EGFR/HER1 and HER2 receptors	Partly unknown	Increased adipocyte lipolysis	(139)
Trastuzumab	Inhibitor of HER2 receptors	Increased lipogenesis	Increased FAS promoter activity	(140)
Tamoxifen	Inhibitor of oestrogen receptors (ERs)	Increased lipogenesis	Increased cholesterol pathway gene expression	(141)

### Fatty Acid Oxidation

Fatty acid β-oxidation (FAO) is a primary bioenergetic source by which fatty acids are broken down in a multistep process. Fatty acids enter cells through fatty acid transport proteins like: fatty acid translocase (FAT/CD36), tissue-specific fatty acid transport proteins (FATP), and plasma membrane-bound fatty acid-binding protein (FABPpm) (142, 143). In breast cancer loss-of-function studies have demonstrated that CD36 is critical in fatty acid uptake (144). Compared to CD36, FATPs and FABPpm have received far less attention (145).

Fatty acid transportation across the mitochondrial membrane is controlled by several enzymes. Once inside the cell, a CoA group is added to the fatty acid by acyl-CoA synthetase, forming long-chain acyl-CoA (146). The conversion of the long-chain acyl-CoA to long-chain acylcarnitine by carnitine palmitoyltransferase 1 (CPT1) is the step by which fatty acid is transported across the inner mitochondrial membrane, the process controlled by carnitine translocase (CAT). CPT2 enzyme converts back the long-chain acylcarnitine to long-chain acyl-CoA that enters the fatty acid β-oxidation pathway (146) (**Figure 4**).

The transport of fatty acyl-CoAs into the mitochondria by CPT1 proteins is considered a rate-limiting step in the mitochondrial fatty acid β-oxidation pathway is (147). All three known isoforms of CPT1 enzyme (CPT1A, CPT1B, and CPT1C) have been recognized as important players in drug resistance. Using an integrated genomic strategy, Gatza ML et al. identified CPT1A as an important player also in cell proliferation especifically in hormone receptor-positive breast carcinomas (148).

The β-oxidation is a cyclic process responsible for the mitochondrial disintegration of long-chain acyl-CoA to acetyl-CoA. This process is controlled by following four main enzymes: acyl-CoA dehydrogenase, enoyl-CoA hydratase, 3-hydroxyacyl-CoA dehydrogenase, and ketoacyl-CoA transferase (thiolase) (149). In each cycle, FAD-dependent dehydrogenation and NAD-dependent oxidation leads to the formation of FADH_2_ and NADH.

Complete oxidation of the produced acetyl-CoA, NADH, and FADH_2_ is accomplished by the TCA and OXPHOS (**Figure 4**) (146, 150).

Many types of cancer exhibit a high activity of FAO such as TNBC, *KRAS* mutant lung cancer, hepatitis B-induced hepatocellular carcinoma etc. (136, 151, 152). The mechanisms of FAO activation in different tumor cells are under serious debate and different mechanisms have been proposed to explain drug-induced FAO activation and the role played by FAO in drug resistance (153).

Studies have reported that prominent oncoproteins play important role in the activation of several FAO enzymes. Overexpression of c-Myc in TNBC resulted in FAO enzymes and metabolic intermediates upregulation whereas inhibition of FAO blocked Myc-driven tumorigenesis (154). Another study identified CPT1B as a downstream target of the JAK/STAT3 pathway in breast cancer (135, 136).

Hoy AJ et al. discuss several aspects of the association of tumor fatty acid metabolism and therapy resistance. In recurrent breast carcinomas they described enhanced CPT1B mRNA expression compared to tumours that did not recur (135). In patients with pancreatic and gastric cancers higher CPT1A expression was associated with chemoresistance and with shorter overall survival (135, 155, 156).

Studies suggest that pathways like PI3K/AKT/mTOR, JAK/STAT3 play a pertinent role in lipid metabolism regulation (157–159). Wang T et al. have found that leptins upregulate STAT3 and FAO activity and this metabolic switch promotes cancer stemness and chemoresistance. In *in vivo* conditions blocking FAO re-sensitized resistant cells to chemotherapy (136, 159). Another study described that inhibition of FAO by mercaptoacetate and etomoxir sensitizes paclitaxel-resistant lung adenocarcinoma cells (160).

Targeting FAO came into the focus of researches as a chemosensitization strategy given its key role in promoting tumor cell survival *via* energy generation. More and more studies suggest that addition of FAO inhibitors completely or partially inhibited drug resistance of cancer cells (160–162).

### Fatty Acid Biosynthesis

Substantial efforts have been documented to develop strategies to target fatty acid biosynthesis since new and new studies have demonstrated that activation of *de novo* fatty acid synthesis is specific to some cancerous tissues (163).

**Figure 5:**
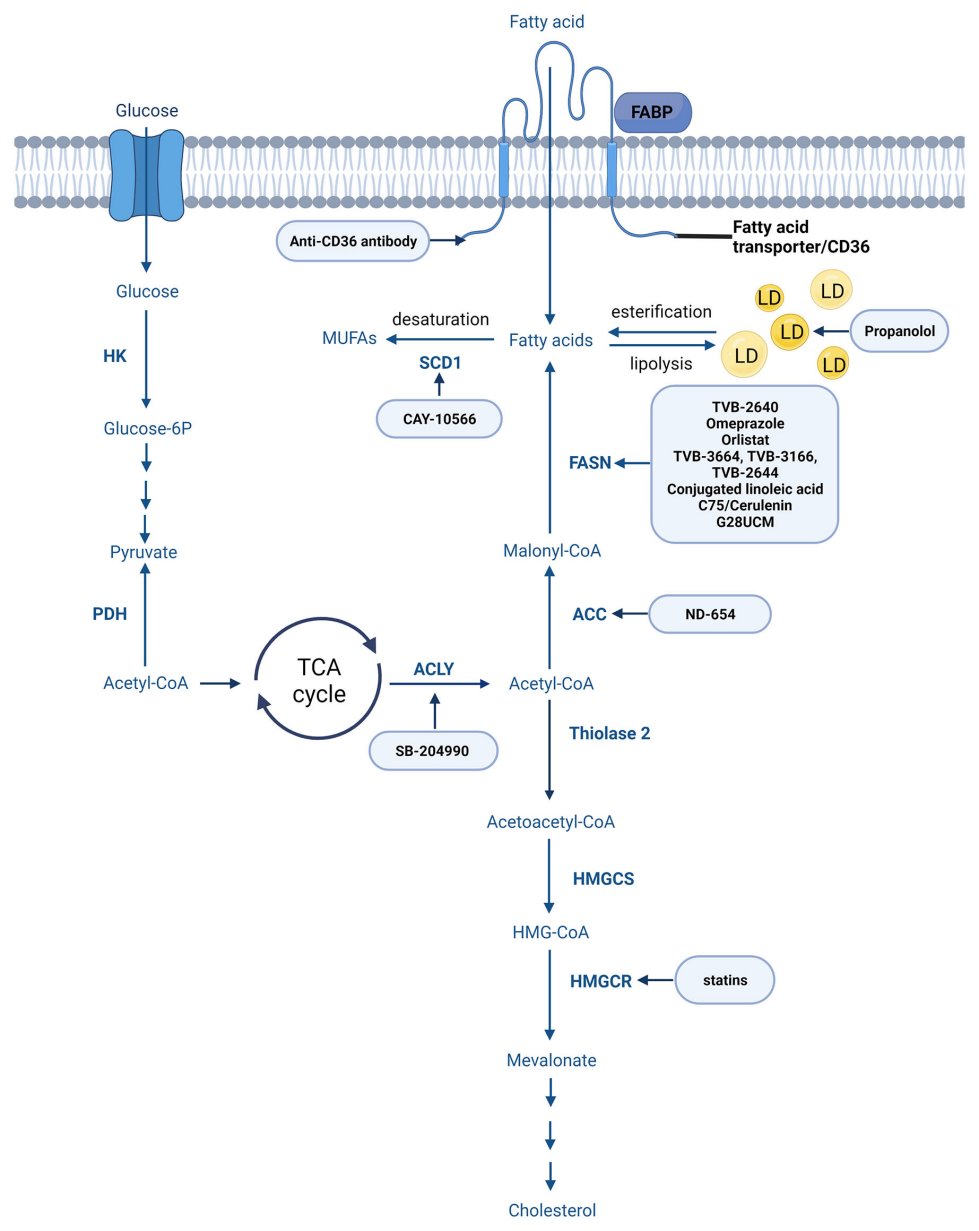
Main steps of fatty acid and cholesterol synthesis. Created with BioRender.com. Agreement number: YB23MRDXCR.

In a standard way fatty acids are synthesized through the fatty acid synthesis cycle (**Figure 5**). The substrate for FA synthesis is cytoplasmic acetyl-CoA, which is obtained through different mechanisms (163). The synthesis of fatty acids from acetyl-CoA and malonyl-CoA involves two main steps: 1. carboxylation of acetyl-CoA by acetyl-CoA carboxylase (ACC) to form malonyl-CoA (ATP dependent) and 2. decarboxylation of the malonyl group in the condensation reaction catalyzed by the multifunctional FASN containing substrate shuttling domain ACP and six catalytic domains: malonyl/acetyl-CoA-ACP transacylase, β-ketoacyl-ACP synthase, β-ketoacyl-ACP reductase, β-hydroxyacyl-ACP dehydrase, enoyl-ACP reductase and thioesterase (163, 164).

The enzymes playing role in FA biosynthesis are under the control of sterol regulatory element-binding proteins (SREBPs) (165). The three isoforms of SREBPs (SREBP1a, -1c, and -2) are encoded by two different genes (166). The importance of SREBP-1 in lipid metabolism and tumor prognosis is discussed in several papers (167, 168). *In vitro* studies performed on MDA-MB-231 and MCF7 breast cell lines revealed that the suppression of SREBP-1 significantly inhibited cell migration and invasion (168). Targeting SREBP could therefore be an efficient strategy to halt tumor growth (163). Fatostatin, a nonsteroidal diarylthiazole derivative inhibits the activation of SREBP1 and additionally inhibits cell proliferation (169).

#### FASN

Overexpression of FASN is considered as one of the major changes in lipid metabolism associated with drug resistance. FASN is overexpressed in breast carcinomas with over 70% of primary TNBC and in several other epithelial malignancies as demonstrated with immunohistochemistry. Breast cancers overexpressing FASN are more likely to recur and metastasize and present significantly shorter disease-free and overall survival (170, 171). FASN induces resistance to multiple DNA-damaging agents including DOX and cisplatin (170). The impact on sensitivity to microtubule agents such as taxane is contradictory. Sardesai SD et al. have found that FASN induces resistance to multiple DNA-damaging agents including DOX and cisplatin without impacting sensitivity to microtubule agents or antimetabolites whereas Menendez JA et al. have found that the inhibition of FASN activity strengthens the cytotoxicity of docetaxel in the HER2-overexpressing breast cancer cell lines (170, 171). FASN mediates drug resistance *via* palmitate production involving different pathways. Palmitate limits AC transmembrane uptake by cancer cells by modulating the transmembrane mobility of the drugs, a major way for AC entry inside the cells (16). FASN overexpression is also associated with the suppression of drug (DOX)-induced ceramide production by inhibiting the activity of sphingomyelinase (172).

Moreover FASN is an established therapeutic target. Wang W et al. in a recent study detailed the new therapeutic perspectives in cancers based on the lipid metabolic pathway (173).

Summarized data of the therapeutic challenges in lipid metabolism are presented in **Table 4**.

**Table 4:** Non-exhaustive list of drugs targeting lipid metabolism used in association with standard treatments in resistant cancer.

Target	Drug name	Drug effect	Drug Combination	References/trial number
FASN	Orlistat	Pancreas lipase inhibitor anti obesity drug approved by FDA	Trastuzumab, Taxanes	(174)
	TVB-3664, TVB-3166, TVB-2644	Reversible and selective FASN inhibitor		(175, 176)
	Omeprazole	Proton pump inhibitor		(173)
	Conjugated linoleic acid	Reduces FASN gene expression		(173)
	C75/Cerulenin	Inhibition of β-ketoacyl-synthase activity	Trastuzumab	(177)
	G28UCM	Selective FASN inhibitor	Trastuzumab, Lapatinib, Gefitinib, Erlotinib	(178)
FACS	Triacsin C	Inhibitor of fatty acyl-CoA synthetase 1and 4	Paclitaxel, Doxorubicin	(179, 180)
HMG-CoA reductase	Statins	Inhibitors of HMG-CoA reductase	Doxorubicin, Daunorubicin	(181, 182)
FPTase	L-744,832	Selective inhibitor of FPTase	Doxorubicin	(183)
Lipin	Propanolol	Inhibition of Lipin-1	Rapamicin	(184)
FAT/CD36	Anti-CD36 antibody	Irreversible inhibition of CD36	Tamoxifen	(185)
CPT1/CPT2	Perhexiline	CPT1 and 2 inhibitors	Lapatinib	(186)
SCD-1	CAY-10566	Selective SCD-1 inhibitor		(187, 188)

A real challenge in targeting lipid metabolism is the lack of selectivity. For example the first generation of FASN inhibitors such as Orlistat (presenting with lack of selectivity, poor metabolic stability) displayed considerable side effects (e.g. anorexia). The next-generation FASN inhibitors present limited systemic toxicity in a preclinical study, higher anti-tumor potential and higher specificity for FASN (173, 189). TVB-2640, a selective FASN inhibitor showed good tolerability and efficacy when combined with taxol in previously treated patients with advanced metastatic breast cancer. By inhibiting FASN a partial regression in ~20% of patients and stable disease in the remainder of patients was documented (189). Based on the very recent study of Sardesai SD et al. Omeprazole can be safely administered in doses that inhibit FASN. Further validation is needed but a promising pCR rate was diagnosed after the addition of Omeprazole to neoadjuvant AC-T (170).

Other enzymes involved in fatty acid synthesis might also be therapeutic targets in cancer. Inhibition of key enzymes for FAs synthesis, such as FASN (presented above), ATP-citrate lyase (ACLY) and ACC can decrease the proliferation and growth of different cancer cells. In hepatocellular carcinoma, ND-654, an ACC inhibitor, has been shown to suppress carcinogenesis (190). In human glioblastoma cells reduced proliferation and *de novo* lipogenesis was observed after the inhibition of acetyl-CoA carboxylase 1 (ACC1) and 2 (ACC2) (190, 191). However, Jeon SM et al. targeting ACCs by short interfering RNAs (siRNAs) have found that only ACC2 knockdown inhibited markedly cell death and H_2_O_2_ accumulation without decreasing O_2_ (192).

#### ACLY

ACLY, a cytoplasmic enzyme catalyzing citric acid breakdown to acetyl-CoA, was found to be overexpressed in several cancers like breast, colorectal, non-small cell lung cancers etc. (193). The ACLY inhibitor SB-204990 has been set out to inhibit the proliferation of lung adenocarcinoma cells *in vivo* and *in vitro.* Other ACLY inhibitors like difluorocitric acid and hydroxycitrate have been demonstrated to block the synthesis of FAs. Although some ACLY inhibitors performed well and have been validated, most of the studies to date are still in the preclinical phase (193–195).

Mitochondrial proteomics reveal acetyl-CoA acetyltransferase, hydroxacyl-CoA dehydrogenase and short chain enoyl-CoA hydratase overexpression in DOX-resistant compared to DOX-sensitive uterine sarcoma cells, providing potential diagnostic markers and therapeutic candidates (16).

#### SCD1

It is worth mentioning the stearoyl-CoA desaturase 1 (SCD1) enzyme as another rate-limiting enzyme in fatty acid synthesis that converts saturated acids to monounsaturated fatty acids (MUFAs) involved in many biological processes (are major constituent of biological structures such as membranes and can also function as or modify signaling molecules). Accordingly more and more studies highlight different roles of SCD1 like modulation of autophagy (196) others discussing the involvements of SCD1 in regulation of cancer stem cells (197) and in the promotion of cancer cell proliferation, migration and metastasis (198). Cancer cells presenting with high degree of membrane saturation are known to be less sensitive to oxidative stress induced by agents like ACs. It is intriguing and important to find combination of therapies that acts synergistically. Many inhibitors of SCD1 like CAY10566, MF-438 and CVT-11127 have been presented but only a few have progressed to clinical trials due to their adverse effects (198).

### The Role of Membrane Lipid Composition in Chemotherapy Resistance

Reduced fluidity of lipid bilayers in the membranes are also characteristics of chemoresistant cancer cell lines. Membrane lipid composition has gained high importance in cancer research. The reduced membrane fluidity influence or disrupt drug uptake *via* passive diffusion or endocytosis (135). Plasma membrane cholesterol was reported to be elevated in AC-resistant MCF-7 breast cancer cells (199). The increased expression of sphingolipid metabolizing enzymes, specifically ceramide transport protein, sphingosine kinase 1 and 2 have been correlated with resistance to DOX and paclitaxel (39).

Studying the cholesterol metabolic reprogramming in cancer came in the focus of researches in the last years based in part on its important role in maintaining cellular homeostasis (providing essential hormones) as well as considering its role in forming lipid rafts, an indispensable cell membrane structure in cancer cells. As many tumor-related proteins are located in lipid rafts this cell component is an important platform for oncogenic signaling pathways. Considering other aspects like the metabolites obtained by *de novo* modification of cholesterol (mevalonic acid, farnesyl pyrophosphate and geranylgeranyl pyrophosphate) also playing an important role in cancer growth and/or oncogenic signaling pathways it is reasonable to consider the significant role played by cholesterol in tumor metabolism. Cholesterol deficiency in cell membranes has been shown to inhibit cancer progression (157) and cholesterol metabolic reprogramming in cancer cells is strongly linked to several aspects of drug resistance (157, 200).

Cholesterol is mostly synthetized through the mevalonate pathway (**Figure 5**) or acquired from the circulation *via* LDL receptor (LDLR)-mediated endocytosis. In the mevalonate pathway 3-hydroxy-3-methylglutaryl-CoA (HMG-CoA) is reduced to mevalonate by HMG-CoA-reductase enzyme considered as the primary control site for cholesterol biosynthesis (201). The enzyme activity and protein level is controlled by multiple regulatory mechanisms, its competitive inhibitors are among the most widely prescribed medications, collectively known as statins. Further a series of enzymatic reactions convert mevalonate to cholesterol (199, 202, 203).

The role of cholesterol in drug resistance has been demonstrated in several cancer types including breast cancer (199) and is mostly related to AC resistance given the role of the cell membrane permeability in AC drugs. It was shown that decreased cholesterol levels resulted in the increased uptake of DOX (204).

Some mechanisms through which cholesterol regulates drug resistance are lipid rafts, ABC transporters, drug uptake. These mechanisms are highly discussed in a recently published review of Yan A et al. (199).

The “membrane-lipid therapy” based on the modulation of membrane lipid composition is considered as an effective therapeutic strategy in several diseases. Preta G. mentions that instead of modifying membrane cholesterol/sphingolipids content new schemes like modulation of membrane bilayer properties (fluidity and elasticity) by inducing changes in the organization of lipid rafts are preferred (205).

The accumulation of lipid droplets is a less well-studied aspect of chemoresistant cancer cell lines (135). Lipid droplets are considered to directly contribute to chemoresistance by providing an extra source of lipids for FAO when nutrient stress occurs, or may play a role in hydrophobic drug sequestration (206).

Sirois I et al. in a very impressive study by analyzing a novel MDA-MB-436 cell-based model of chemoresistance characterized by a unique and complex morphologic phenotype with numerous lipid droplets and a set of primary chemoresistant TNBCs have identified several metabolic vulnerabilities. These include a dependence on perilipin family member perilipin 4 (PLIN4), a protein coating of the observed lipid droplets that play important role in fat mobilisation, expressed both in experimental conditions (in the chemoresistant TNBC cells) and in chemoresistant tumors treated with neoadjuvant DOX-based chemotherapy. Their results call attention to a novel mechanism of chemotherapy resistance that may have therapeutic consequences in the treatment of drug-resistant cancer (207).

## Future Challenges in Lipid Metabolism and Breast Cancer Treatment

Based on the above-mentioned data related to lipid metabolism in breast carcinomas there are some intriguing questions to be analyzed in more detail:

1. How breast carcinoma treatment is going to be tailored based on the metabolic phenotypes/subtypes? In a review by Marie E. Monaco analyzing fatty acid metabolism based on mRNA expression data in breast cancer subtypes suggested that significant differences are observed. While the less aggressive, HR+ (luminal) subtypes depend on a balance between *de novo* fatty acid synthesis and oxidation as sources for energy requirements the TNBCs are characterized by overexpression of the genes involved in the utilization of exogenous fatty acids. They suggest that treatments have to be tailored to individual subtypes (147).
2. Lipid metabolism has been difficult to analyze partly due to technical problems. Considering the challenges (tumor heterogeneity, metabolic diversity, the rapidly changing environmental context, differences in nutrients use among different cell types, the cooperative or competitive relationships between cells, etc.) it is questionable how the new technologies could help in delineating the contribution of lipid metabolism to tumor differentiation, progression and resistance to different drugs. Matsushita Y et al. summarize the new technology currently used in lipidomics and confirm that recent innovations especially in mass spectrometry- and chromatography-based lipidomics technologies have improved our understanding of the role of lipids in cancer (208).
3. Cancer-associated adipocytes are poorly investigated cells in breast tumor microenvironment. What should be the role of the surrounding and breast cancer-associated adipocytes? A few studies suggest that cancer-associated adipocytes can also cause resistance to radiotherapy and to chemotherapeutic drugs (209, 210). Another study described that interaction between cancer-associated adipocytes and cancer cells seems to be more pronounced in obese patients (211).
4. How is lipid profile of patients restored after (neo)adjuvant chemotherapy? Tian W et al. suggested that different age groups showed different changes in lipid levels and in general the lipid profiles were restored to baseline levels approximately 6 months after chemotherapy completion (212).

## Conclusions

Main metabolic alterations in breast cancers include the preference of the glycolytic pathway (the increased uptake of glucose, hyperactivated glycolysis), the enhanced oxidative phosphorylation pathway and dysregulation of fatty acid metabolism.

Current studies present the important aspect of tumor metabolism associated with anticancer drug resistance and the association of the partly unknown aspect of the metabolic plasticity in chemotherapy resistance. Efforts are made to combine chemotherapies with drugs targeting different steps of metabolic pathways. Compared to a huge amount of results related to the above-mentioned questions only a few studies present data related to changes in metabolic profile induced by currently applied chemotherapeutic agents used in neoadjuvant or adjuvant settings. There are also gaps in analysing the association of the metabolic changes induced by chemotherapies with different response rates to the applied treatment regimen or with tumor progression.

Considering just some of the points like: the regulation of lipid and glucose metabolism is also important for normal cells, the intra- and intertumoral metabolic heterogeneity in breast cancers, the different metabolic signatures observed in different breast carcinoma subtypes, the lack or imperfect technologies to study components of metabolic pathways, it is still a huge challenge to find substances that target different steps of glucose and lipid metabolism in the very heterogeneous group of breast tumors without affecting normal cells. Additionally, current metabolomic and especially lipidomic approaches have identified several partly unknown components of metabolic pathways paving the way to a better understanding of cancer biology. As exemplified in our and in recently published reviews there are relatively broad opportunities in targeting metabolism in cancers but it is a key question whether we have considered nearly all metabolic hubs as targets and whether we can rewire our mind to optimise the therapies based on the several new data.

## Author Contributions

AMT: conceptualization, interpretation of data, writing original draft, review. JK: review, interpretation of data. SV-K: editing, preparing figures. BT: review, editing, preparing figures and supervision. All authors contributed to the article and approved the submitted version.

## Funding

This study was supported by the CELSA 19/037 grant." FAT-MetBC: Facing Increased Adiposity in Treating Metastatic Breast Cancer.

## Conflict of Interest

The authors declare that the research was conducted in the absence of any commercial or financial relationships that could be construed as a potential conflict of interest.

## Publisher’s Note

All claims expressed in this article are solely those of the authors and do not necessarily represent those of their affiliated organizations, or those of the publisher, the editors and the reviewers. Any product that may be evaluated in this article, or claim that may be made by its manufacturer, is not guaranteed or endorsed by the publisher.

## Glossary

**Table:**

1,3 BPG	1,3 bisphosphoglycerate
2-DG	2-deoxy-D-glucose
2PG	2-phosphoglycerate
HMG-CoA	3-hydroxy-3-methylglutaryl-CoA
ACC1	acetyl-CoA carboxylase 1
ACC	acetyl-CoA carboxylase
AC	anthracylines
AA	arachidonic acid
ACLY	ATP-citrate lyase
3- BrPA	bromopyruvate
CBZ	cabazitaxel
CPT1	carnitine palmitoyltransferase 1
DTX	docetaxel
DOX	doxorubicin
ENO	enolase
FPTase L-744	832 Selective inhibitor of FPTase, farnesyl transferase inhibitor
FA	fatty acid
FAO	fatty acid β-oxidation
FACS	fatty acyl-CoA synthetase
FASN	fatty acid synthase
FAT/CD36	fatty acid translocase
FATP	fatty acid transport proteins
FEC	fluorouracil, epirubicin and cyclophosphamide
FBP	fructose-1,6-bisphosphate
F6P	fructose-6-phosphate
G6PD	glucose-6-phosphate-dehydrogenase
G6P	glucose-6-phosphate
GAPDH	glyceraldehide-3-phosphate dehydrogenase
GAP	glyceraldehyde-3-phosphate
HK	hexokinase
HR	hormone receptor
LD	lipid droplets
LND	lonidamine
LDL	low-density lipoprotein
MDR	multidrug resistance
NeST	neoadjuvant systemic therapy
OXPHOS	oxidative phosphorylation
PTX	paclitaxel
pCR	pathological complete response
PPP	pentose phosphate pathway
PLIN4	perilipin 4
PEP	phosphoenolpyruvate
PFK-1	phosphofructokinase-1
PGI	phosphoglucose isomerase
PGK	phosphoglycerate kinase
PGM	phosphoglycerate mutase
FABPpm	plasma membrane bound fatty acid binding protein
PK	pyruvate kinase
SCD	stearoyl-CoA desaturase
SREBPs	sterol regulatory element binding proteins
TOPOII	topoisomerase II
TCA	tricarboxylic acid cycle
TPI	triose phosphate isomerase
TNBC	triple-negative breast cancer
